# The Relationship Between Parent‐Child Attachment and Peer Attachment and Depression in College Students: A Moderated Polynomial Regression With Response Surface Analyses

**DOI:** 10.1002/pchj.70052

**Published:** 2025-09-21

**Authors:** Ya‐xuan Qin, Hai‐yue Li, Jia‐Yi Zhou, Jun‐ying Han, Yi‐jia Li, Gui‐xiang Tian, Yi Wang, Yan‐yu Wang

**Affiliations:** ^1^ School of Psychology Shandong Second Medical University Weifang China; ^2^ Neuropsychology and Applied Cognitive Neuroscience Laboratory, CAS Key Laboratory of Mental Health, Institute of Psychology Chinese Academy of Sciences Beijing China; ^3^ Department of Psychology University of Chinese Academy of Sciences Beijing China

**Keywords:** attachment, congruence, depression, gender differences, response surface analysis

## Abstract

Previous research has established a strong link between parental attachment and depression in youth. However, the nuances of paternal‐maternal attachment congruence and its relationship with depressive symptoms, as well as the roles of gender differences and peer attachment in this context, remain unclear. This study aimed to explore these associations among emerging adults. Attachment and depressive symptoms were assessed in 1564 college students using the Inventory of Parent and Peer Attachment (IPPA) and the Patient Health Questionnaire‐9 (PHQ‐9), respectively. Polynomial regression and response surface analysis were utilized for data analysis. The results revealed that when paternal and maternal attachment were congruent, students with average‐range levels of parental attachment (i.e., scores near the IPPA mean) exhibited the least depressive symptoms. Conversely, greater discrepancies between paternal and maternal attachment were associated with more pronounced depressive symptoms, while this effect was buffered by higher levels of peer attachment. In addition, incongruent paternal and maternal attachments were not significantly associated with depressive symptoms in sons, whereas insecure maternal attachment was more closely related to daughters' depressive symptoms. In conclusion, this study highlights the importance of parental attachment (in)congruence in college students' depressive symptoms and the moderating roles of gender and peer attachment.

## 1 Introduction

The transition from late adolescence to early adulthood places college students at elevated risk of depression (Liu et al. 2022). According to the Report on National Mental Health Development in China (2021–2022), the depression detection rate among individuals aged 18 to 24 is 24.1%, which is significantly higher than that of other age groups (Fu et al. 2023). Interpersonal perspectives on depression emphasize that maladaptive behaviors in social interactions, particularly negative interactions with significant others, are key contributors to the development and maintenance of depressive symptoms (Brown and Harris 1978; Marchand‐Reilly 2009). According to attachment theory, from infancy, individuals gradually establish deep and lasting emotional bonds with their parents through continuous interaction (Ainsworth and Bowlby 1991). A positive relationship with caregivers who are always sensitive and responsive plays a crucial role in an individual's mental health, while inconsistent caregiver sensitivity increases the likelihood of insecure attachment and depression (Dagan and Sagi‐Schwartz 2018; Roepke and Seligman 2016).

Although parents and children can form parent‐child attachment due to frequent interactions, there is still some debate over whether the father's or the mother's influence on the child is greater (Ainsworth et al. 2015; Dagan and Sagi‐Schwartz 2018). The independence hypothesis suggests that a single primary attachment figure typically exists, usually the mother, as the primary caregiver, playing a central role in shaping the attachment style that influences the child's developmental trajectory, while the impact of other caregivers is relatively minor (Duchesne and Ratelle 2014; Tamura 2018). The integrative hypothesis suggests that parental roles in attachment development are complementary. Mothers tend to provide care and comfort, while fathers are more likely to foster autonomy and encourage confident exploration of novel situations (Duchesne and Ratelle 2014). Both are considered equally crucial (Lunkenheimer et al. 2020).

To better clarify the association between paternal‐maternal attachment and individual mental health (Dagan and Sagi‐Schwartz 2018; Matetovici et al. 2025; Wong et al. 2019), Dagan and Sagi‐Schwartz (2018) further classified parent‐child attachment into congruent (high paternal attachment and high maternal attachment, HH; low paternal attachment and low maternal attachment, LL) and incongruent (high paternal attachment and low maternal attachment, HL; low paternal attachment and high maternal attachment, LH) categories based on the degree of attachment relationships with each parent (Dagan and Sagi‐Schwartz 2018). Some of the results revealed that individuals demonstrating congruent secure attachment to both parents (HH) exhibit more favorable developmental outcomes compared to those with incongruent attachment (HL/LH) (Dagan and Sagi‐Schwartz 2018; Kennison and Spooner 2020). For example, Kochanska and Kim (2013) found that children securely attached to both parents reported fewer internalizing problems than those securely attached to only one parent, while those with dual insecure attachment reported the highest levels. However, there are also studies suggesting that secure attachment to one parent can moderate the risk effects of insecure attachment to the other parent (Dagan et al. 2021). That is, there may be no significant advantage to having a congruent secure attachment relationship with both parents compared to an incongruent situation (Dagan et al. 2021). Therefore, whether their congruency is linked to individuals' depressive symptoms remains unclear.

Additionally, an increasing number of studies have begun to focus on the intergenerational influence of gender roles on the relationship between parent‐child attachment and individual depression (Guttmann and Rosenberg 2003; Riley 2024). Some studies have shown that mothers are more likely to form secure attachments with daughters than with sons, which has a stronger influence on daughters' psychological well‐being (Schoppe‐Sullivan et al. 2006; Tamura 2018). Similarly, paternal attachment has been found to predict depressive symptoms in sons (Pan et al. 2017). However, other studies argue that the influence of the opposite‐sex parent may be more pronounced. For instance, some studies have found that the father's absence had a greater impact on daughter's internalizing problems during childhood (Castetter 2020; Culpin et al. 2013; Ramatsetse and Ross 2022). Overall, these findings suggest that a gender‐matching effect may exist in the association of maternal and paternal attachment with children's mental health. However, it remains uncertain if this effect is present among college students and how it may vary with depressive symptoms across genders.

According to the systematic perspective of the family and peer system, attachment relationships formed by children with multiple figures, such as parents and peers, interact to shape an individual's cognitive and emotional development (Brown and Bakken 2011; Zou et al. 2020). For college students in early adulthood, on the one hand, the parent‐child relationship patterns established in early childhood continue to play a crucial role in their mental health (Shah et al. 2023). On the other hand, the increasing demand for autonomy and independence, coupled with prolonged separation from their families for educational purposes (Arnett 2007), results in a gradual weakening of the influence of parental attachment (Zou et al. 2020). These transitional dynamics may lead to a shift in their attachment toward peers and intimate relationships (Manvelian et al. 2023). Some studies view peer attachment as an extension of parental attachment (Delgado et al. 2022; Sun et al. 2025), suggesting that parental attachment security may shape peer attachment security (Pallini et al. 2014) and that peer attachment mediates the relationship between parental attachment and adolescent depression (Sun et al. 2025). In contrast, others argue that distinct attachment patterns exist between the two, suggesting a complementary relationship (Santiago et al. 2017; Yang et al. 2022). Secure peer attachment has been shown to buffer the negative effects of insecure parental attachment, reducing the chances of internalizing problems (Santiago et al. 2017; Yang et al. 2022). As previously mentioned, there is continuity or discontinuity between parental and peer attachment. It remains unclear whether peer attachment and parental attachment interact to influence depressive symptoms in college students.

**FIGURE 1:**
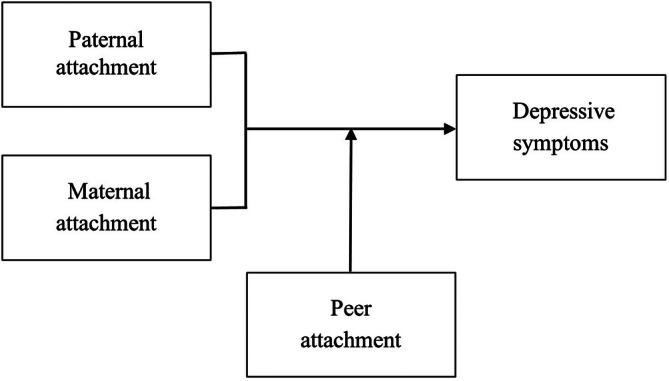
Conceptual framework.

In summary, although previous studies have identified a strong link between parental attachment and depression in children, most have focused on the independent effects of paternal and maternal attachment or have simplified both into a single parental attachment variable to explore its relationship with depression (Dagan and Sagi‐Schwartz 2018; Matetovici et al. 2025; Wong et al. 2019). Research on how paternal‐maternal attachment (in)congruence relates to depressive symptoms of sons and daughters, as well as the moderating effect of peer attachment in this relationship, remains unclear. Methodologically, prior research often used difference scores to examine the effects of the congruence between two variables on an outcome variable (Xu and Zheng 2022). However, this approach may lead to several limitations, including dimensional reduction, decreased reliability, and difficulties in interpreting regression coefficients (Edwards 2002; Hou et al. 2020). To explore these issues, the present study utilized polynomial regression and response surface analysis to examine the relationship between paternal‐maternal attachment (in)congruence and depressive symptoms among college students. Drawing on previous findings and relevant theoretical frameworks (Dagan and Sagi‐Schwartz 2018; Dagan et al. 2021; Edwards 2002; Shanock et al. 2010), the following hypotheses were proposed: (1) When paternal and maternal attachment are incongruent, a greater discrepancy between the two is associated with more severe depressive symptoms. (2) A gender‐matching effect exists in the relationship between paternal and maternal attachment and depressive symptoms, such that maternal attachment is more closely related to depressive symptoms of daughters. (3) Peer attachment moderates the relationship between incongruent parental attachment and depressive symptoms among college students. The conceptual framework for this is shown in Figure 1.

## 2 Method

### 2.1 Participants and Procedure

This cross‐sectional study utilized data collected in December 2023. The minimum required sample size was determined using the standard formula for proportion estimation:

*N* = Z^2^_*α*/2_× [P (1 − *P*)]/δ^2^, where Z_*α*/2_ = 1.96 (i.e., corresponding to a 95% confidence level), δ = 0.03 (i.e., margin of error), and *p* = 0.241 (the prevalence of depression among Chinese university students reported by Fu et al. 2023). Based on these parameters, the calculated minimum sample size was 781 participants.

Prior to completing the online questionnaire, participants were informed about the principles of anonymity and voluntariness. Moreover, to ensure response validity, several lie‐detection items were included in the questionnaire. A total of 1564 valid responses were collected, including 808 male students (51.66%) and 756 female students (48.34%). The participants' ages ranged from 18 to 23 years, with a mean age of 19.77 ± 0.89 years. The study was approved by the Ethics Committee of Shandong Second Medical University (2023YX080), and all participants provided written informed consent.

### 2.2 Measurement

#### 2.2.1 The Inventory of Parent and Peer Attachment (IPPA)

The Chinese version of the Inventory of Parent and Peer Attachment (IPPA) (Armsden and Greenberg 1987; Zhang et al. 2011) was used to assess college students' attachment. This version contains 75 items equally divided into the three forms for mother (e.g., “My mother respects my feeling”), father (e.g., “My father respects my feeling”) and peers (e.g., “I like to get my friend's point of view on things I'm concerned about”). Participants rated each question on a scale from 1 to 5 (1 = *strongly disagree*, 5 = *strongly agree*) by considering their closest maternal figure (*α* = 0.892), closest paternal figure (*α* = 0.904), and close friends (*α* = 0.926). Higher scores reflect a more secure attachment.

#### 2.2.2 The Patient Health Questionnaire‐9 (PHQ‐9)

The Chinese version of the Patient Health Questionnaire‐9 (PHQ‐9) (Kroenke et al. 2001; Zhang et al. 2013) was used to evaluate depressive symptoms among college students (e.g., “Little interest or pleasure in doing things”). All items are rated on a 4‐point scale (0 = *not at all* to 3 = *nearly every day*), with higher scores indicating higher levels of depressive symptoms. In this study, the Cronbach's *α* for the PHQ‐9 scale was 0.*905*.

### 2.3 Analysis

Descriptive statistics and correlation analyses were performed first using SPSS 23.0. Then, polynomial regressions in combination with response surface analysis (RSA) using R software (R version 4.3.1, RSA package version 0.10.6) were conducted to examine the association between paternal‐maternal attachment (in)congruence and depressive symptoms in college students (Shanock et al. 2010).

First of all, paternal attachment and maternal attachment were centered around the pooled grand mean to reduce multicollinearity before calculating the second‐order terms (Edwards 2002). Age, gender, and sibling status were included as control variables in the model. Depressive symptoms (Z) were then regressed on the control variables, centered paternal attachment (X), centered maternal attachment (Y), paternal attachment squared (X^2^), paternal × maternal attachment (XY), and maternal attachment squared (Y^2^). The equation applied was as follows (to simplify the equation, control variables were omitted): Z = b_0_ + b_1_X + b_2_Y + b_3_X^2^ + b_4_XY + b_5_Y^2^ + e. If *R* ^2^ was significant, it indicated that the model could explain variations in depressive symptoms. Four coefficients (a_1_, a_2_, a_3_, and a_4_) were then evaluated to capture the three‐dimensional relationship among X, Y, and Z. The congruence line (X = Y) and the incongruence line (X = −Y) were key features of the response surface. The slope and curvature along the congruence line were represented by a_1_ = (b_1_ + b_2_) and a_2_ = (b_3_ + b_4_ + b_5_), respectively. For the incongruence line, a_3_ = (b_1_ − b_2_) and a_4_ = (b_3_ − b_4_ + b_5_) reflected the corresponding slope and curvature. The response surface, constructed using the regression coefficients, visually illustrated how paternal and maternal attachment jointly influenced depressive symptoms under different levels of attachment (in)congruence.

Finally, to test moderation effects, peer attachment (W) was added as a moderator to the model: Z = b_0_ + b_1_X + b_2_Y + b_3_X^2^ + b_4_XY + b_5_Y^2^ + b_6_W + b_7_WX + b_8_WY + b_9_WX^2^ + b_10_WXY + b _11_WY^2^ + e, if the model's Δ*R* ^2^ was significant after the addition of the moderator term, it indicated that the moderation effect held. A simple effect analysis was then conducted to examine the curvature, slope, significance, and effect size in samples with varying levels of peer attachment.

## 3 Results

### 3.1 Preliminary Analyses

Following previous recommendations (Fleenor et al. 1996), we analyzed group differences using a threshold of 0.5 standard deviations, and all variables were standardized. In the sample, 1122 students (71.74%) reported congruent paternal and maternal attachment, while 442 students (28.26%) reported incongruence between paternal and maternal attachment. Specifically, 195 students (12.47%) exhibited high paternal attachment and low maternal attachment, while 247 students (15.79%) showed low paternal attachment and high maternal attachment. These findings met the basic assumptions for polynomial regression analysis, with each category containing more than 10% of the sample, and reflected the multifaceted nature of parental attachment in college students.

Additionally, as shown in Table 1, correlation analysis revealed significant positive correlations between paternal attachment, maternal attachment, and peer attachment. Furthermore, paternal attachment, maternal attachment, and peer attachment were all negatively correlated with depressive symptoms in college students (*p*s < 0.010).

**TABLE 1:** Descriptive statistics and correlation analysis.

	1	2	3	4
1 Paternal attachment	1			
2 Maternal attachment	0.774**	1		
3 Peer attachment	0.663**	0.617**	1	
4 Depressive symptoms	−0.284**	−0.287**	−0.165**	1
*M*	93.04	96.54	90.61	1.86
SD	18.99	17.59	16.29	3.52

*Note*: Bonferroni correction: *p < 0.05, **p < 0.01, *n* = 1564.

### 3.2 Testing for (In)Congruence Effects on College Students' Depressive Symptoms

**FIGURE 2:**
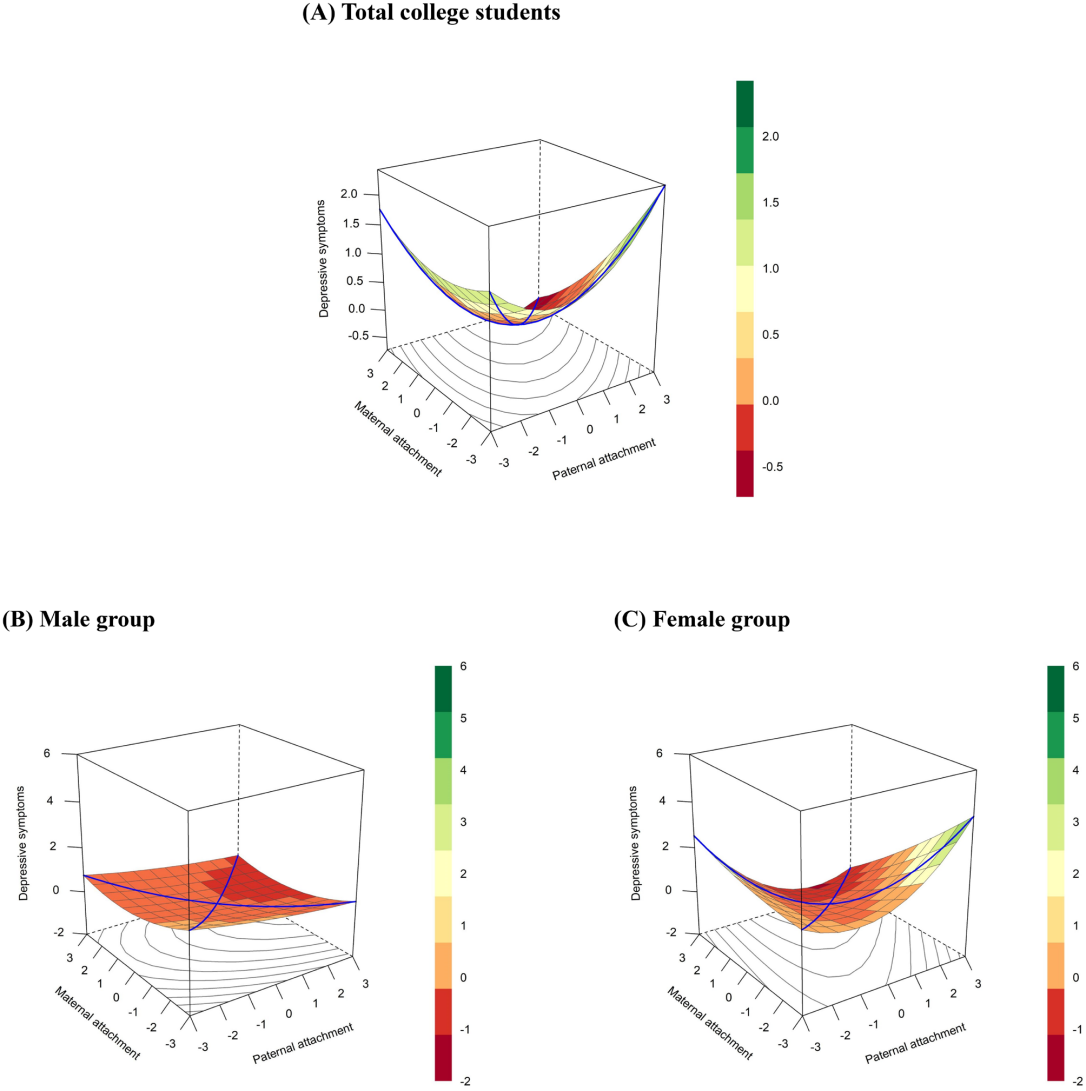
Response surface plots. (A) Plot of total college students. (B) Plot of the male group. (C) Plot of the female group. The rotation position is *x* = −63, *y* = 32, *z* = 15; the color in the response surface indicates the level of outcomes.

The results of the polynomial regression and response surface analysis on the relationship between paternal‐maternal attachment (in)congruence and depressive symptoms in college students are presented in Table 2. The slope of the congruence line (X = Y) was significantly negative (a_1_ = −0.34, 95% CI [−0.40, −0.29], *p* < 0.001), indicating that students with lower levels of both paternal and maternal attachment experienced higher depressive symptoms compared to those with higher levels of both. The curvature of the congruence line (X = Y) was significantly positive (a_2_ = 0.06, 95% CI [0.01, 0.11], *p* = 0.021), suggesting a nonlinear relationship between congruent parental attachment and depressive symptoms. When paternal and maternal attachment were congruent, whether at low or high levels of attachment, college students exhibited higher levels of depressive symptoms. Combined with the response surface plot (Figure 2A), it can be seen that the degree of depressive symptoms initially decreased from the highest point (Z_LL_) to an inflection point, then increased again toward the second‐highest point (Z_HH_). Students with average‐range levels of both paternal and maternal attachment exhibited the lowest degree of depressive symptoms.

**TABLE 2:** Polynomial regressions of college students' depressive symptoms on attachment congruence/incongruence.

Variables	Depressive symptoms		
	Total (*n* = 1564)	Male students (*n* = 808)	Female students (*n* = 756)
b_1_‐X	−0.12** (95% CI: −0.20, −0.04)	−0.17** (95% CI: −0.29, −0.04)	−0.07 (95% CI: −0.18, 0.05)
b_2_‐Y	−0.23*** (95% CI: −0.31, −0.14)	−0.12 (95% CI: −0.25, 0.00)	−0.33*** (95% CI: −0.46, −0.20)
b_3_‐X^2^	0.11** (95% CI: 0.04, 0.18)	0.01 (95% CI: −0.08, 0.11)	0.19*** (95% CI: 0.09, 0.29)
b_4_‐XY	−0.09* (95% CI: −0.18, −0.01)	0.00 (95% CI: −0.14, 0.14)	−0.17** (95% CI: −0.28, −0.05)
b_5_‐Y^2^	0.04 (95% CI: −0.02, 0.11)	0.06 (95% CI: −0.06, 0.19)	0.03 (95% CI: −0.05, 0.10)
a_1_	−0.34*** (95% CI: −0.40, −0.29)	−0.29*** (95% CI: −0.36, −0.22)	−0.40*** (95% CI: −0.48, −0.32)
a_2_	0.06* (95% CI: 0.01, 0.11)	0.08* (95% CI: 0.00, 0.16)	0.05 (95% CI: −0.02, 0.11)
a_3_	0.11 (95% CI: −0.05, 0.27)	−0.04 (95% CI: −0.28, 0.19)	0.27* (95% CI: 0.04, 0.49)
a_4_	0.24** (95% CI: 0.08, 0.41)	0.08 (95% CI: −0.20, 0.35)	0.38*** (95% CI: 0.16, 0.60)

*Note*: a_1_ = the slope of the congruence line; a_2_ = the curvature of the congruence line; a_3_ = the slope of the incongruence line; a_4_ = the curvature of the incongruence line. X, paternal attachment; Y, maternal attachment. *p < 0.05, **p < 0.01, ***p < 0.001.

At the same time, the slope of the incongruence line (X = −Y) was not significant (a_3_ = 0.11, 95% CI [−0.05, 0.27], *p* = 0.186), indicating that there was no significant asymmetric difference along the incongruence line. The curvature of the paternal‐maternal attachment incongruence line (X = −Y) was significantly positive (a_4_ = 0.24, 95% CI [0.08, 0.41], *p* = 0.004), suggesting that greater discrepancy between paternal and maternal attachment was associated with more severe depressive symptoms in college students (Figure 2A).

### 3.3 Moderating Effect of Gender

Independent sample *t*‐tests indicated a significant difference in depressive scores between male and female students, with male students exhibiting a significantly lower degree of depressive symptoms than female students (*t* = −4.33, *p* < 0.001, *d* = 0.18). A group analysis of the congruence between paternal and maternal attachment showed that more than 10% of both male and female students perceived incongruence. Given this proportion, the current data are suitable for subsequent response surface analysis.

For male students, the slope (a_3_ = −0.04, 95% CI [−0.28, 0.19], *p* = 0.718) and curvature (a_4_ = 0.08, 95% CI [−0.20, 0.35], *p* = 0.579) of the incongruence line were not significant (see Table 2), indicating no significant asymmetric differences along the incongruence line. That is, the effect of paternal attachment on male students' depressive symptoms was not significantly different from that of maternal attachment. In other words, no gender matching effect was observed in male students' families (see Figure 2B). However, for female students, both the slope (a_3_ = 0.27, 95% CI [0.04, 0.49], *p* = 0.021) and curvature (a_4_ = 0.38, 95% CI [0.16, 0.60], *p* < 0.001) of the incongruence line were significant (see Table 2). The results indicated that the degree of incongruence was positively associated with female students' depressive symptoms. In addition, the effect of the direction of incongruence showed that when female students perceived a more insecure maternal attachment than paternal attachment, they tended to exhibit higher depressive symptoms. Consequently, a gender matching effect was apparent in the family dynamics of female students, as depicted in Figure 2C.

### 3.4 Testing for the Moderated (In)Congruence Effects on College Students' Depressive Symptoms

As is shown in Table 3, Δ*R* ^2^ was significant in the third step (Δ*R* ^2^ = 0.03, *p* < 0.001, Cohen's *f* ^*2*^ = 0.04), indicating a moderating effect of peer attachment in this model. Importantly, when analyses were conducted separately for male and female students, the moderating effect of peer attachment remained significant in both subgroups, even after excluding outliers (see Supporting Information). For clarity, we analyzed the slopes and curvatures of the congruence and incongruence lines at low (−1 SD below the mean) and high (1 SD above the mean) levels of attachment.

**TABLE 3:** Hierarchical regression predicting psychological health from paternal‐maternal attachment congruency and peer attachment.

Variables	Δ*R* ^2^	*β*	SE	*t*
First step				
Age		−0.07**	0.02	−2.99
Gender		0.14***	0.03	5.71
Sibling		−0.04	0.02	−1.89
Second step	0.12***			
Paternal attachment (X)		−0.10*	0.05	−2.21
Maternal attachment (Y)		−0.27***	0.05	−5.67
X^2^		0.13***	0.04	3.61
XY		−0.03	0.05	−0.61
Y^2^		0.06	0.03	1.85
Third step	0.03***			
Peer attachment (W)		−0.00	0.04	−0.03
WX		−0.10*	0.05	−2.16
WY		−0.06	0.05	−1.15
WX^2^		−0.11***	0.03	−3.39
WXY		0.17***	0.05	3.79
WY^2^		−0.01	0.03	−0.36
R^2^	0.17***			
*F*	21.80***			

*Note: n* = 1564, *p < 0.05, **p < 0.01, ***p < 0.001.

**FIGURE 3:**
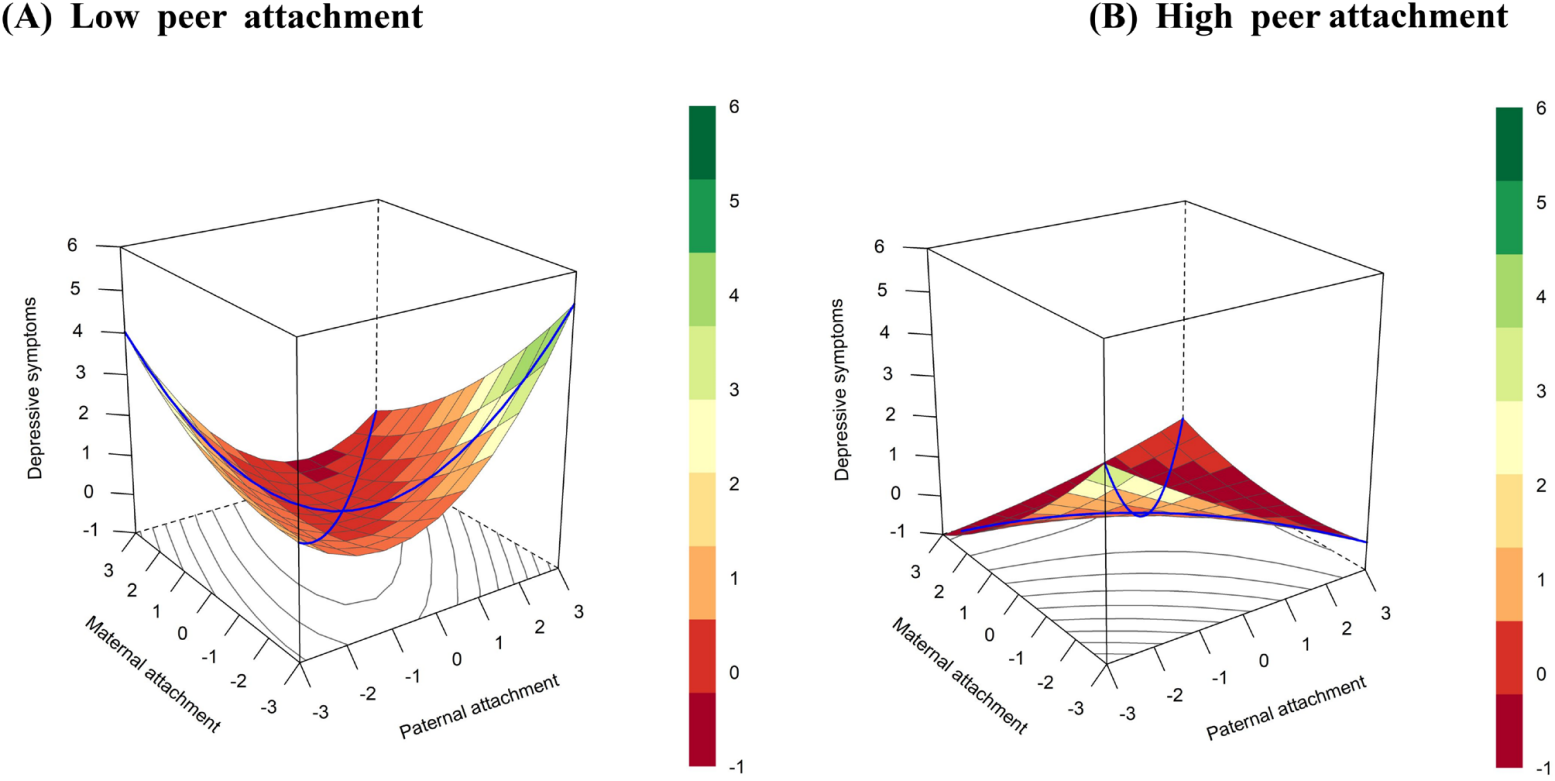
Response surface plots. (A) Plot of the low peer attachment group. (B) Plot of the high peer attachment group. The rotation position is *x* = −63, *y* = 32, *z* = 15; the color in the response surface indicates the level of outcomes.

As Table 4 shows, the slopes and curvatures of the congruence and incongruence lines exhibited different patterns at different levels of peer attachment. Specifically, at the low levels of peer attachment, the curvature of the incongruence line (X = −Y) was significantly positive (a_4_ = 0.52, 95% CI [0.25, 0.79], *p* < 0.001), indicating that greater discrepancy between paternal and maternal attachment was associated with more severe depressive symptoms. However, at high levels of peer attachment, the curvature of the incongruence line (X = −Y) was not significant (a_4_ = −0.07, 95% CI [−0.29, 0.14], *p* = 0.501). This suggests that high peer attachment buffered the relationship between parental attachment incongruence and depressive symptoms. The response surface plot is provided to illustrate the results of the polynomial regression (see Figure 3).

**TABLE 4:** Slopes and curvatures of the in/congruence lines at low/high levels of peer attachment.

Depressive symptoms	Congruence line (X = Y) slope a_1_	Congruence line (X = Y) curvature a_2_	Incongruence line (X = −Y) slope a_3_	Incongruence line (X = −Y) curvature a_4_
Low peer attachment	−0.21***	0.12***	0.21	0.52***
(−1 SD)	(95% CI: −0.32, −0.11)	(95% CI: 0.05, 0.19)	(95% CI: −0.06, 0.48)	(95% CI: 0.25, 0.79)
High peer attachment	−0.53***	0.21***	0.12	−0.07
(+1 SD)	(95% CI: −0.62, −0.44)	(95% CI: 0.13, 0.29)	(95% CI: −0.09, 0.33)	(95% CI: −0.29, 0.14)

*Note: n* = 1564, *p < 0.05, **p < 0.01, ***p < 0.001.

## 4 Discussion

This study utilized polynomial regression and response surface analysis to examine the relationship between paternal‐maternal attachment (in)congruence and depressive symptoms among college students, as well as the moderating roles of gender and peer attachment. The findings revealed that when paternal and maternal attachment were congruent, students with average‐range levels of parental attachment exhibited the least depressive symptoms. In cases of incongruence, a greater discrepancy between paternal and maternal attachment was linked to higher levels of depressive symptoms. Although no significant difference was found between the relationships of paternal and maternal attachment with depressive symptoms in sons, maternal attachment insecurity had a more pronounced effect on daughters' depressive symptoms compared to insecure paternal attachment. Additionally, high levels of peer attachment were identified as a protective moderating factor in the relationship between incongruent parental attachment and depressive symptoms.

Interestingly, our research found that college students who exhibited a balance between attachment security and insecurity (those with average‐range scores on the IPPA scale) reported relatively lower levels of depressive symptoms (Bishop et al. 2018). While previous studies have identified associations between attachment security and depression in children and adolescents (Spruit et al. 2020), our study further examines the role of paternal‐maternal attachment congruence among emerging adults. Arnett (2007) defined emerging adulthood (ages 18–25) as a transitional phase from adolescence to young adulthood, during which excessive emotional dependence on parents may hinder the development of social competencies and increase the risk for both externalizing and internalizing problems (LaFreniere 2024). Additionally, as the core construct of Bowen's family systems theory, self‐differentiation plays a crucial role in balancing emotional attachment and autonomy (Calatrava et al. 2022). Individuals with high self‐differentiation can balance rationality and emotion, as well as experience intimacy and autonomy in relationships with others, whereas excessive involvement in parent‐child triangulation may lower differentiation, leading to greater social avoidance and distress (An et al. 2018), which may further reinforce compensatory overreliance on parental figures, creating a maladaptive feedback loop (Shah et al. 2023). Therefore, secure‐but‐autonomy‐supportive parental attachment appears more protective against depressive symptoms in college students than either overinvolved or detached parental patterns (Jiao and Segrin 2022).

Regarding the incongruence between paternal and maternal attachment, it was associated with higher depressive symptoms in college students. This finding supports the spillover hypothesis, which proposes that individuals' affect and behaviors can transfer between family subsystems (Martin et al. 2017; Zou et al. 2020). High levels of congruent paternal‐maternal attachment may reflect positive parental interaction (Feinberg 2003; Zou et al. 2020), which can buffer against depressive symptoms (Riina and McHale 2014). Conversely, greater incongruence may indicate family dysfunction (Lee et al. 2015), often marked by intense conflict or separation (Kozlowska et al. 2021). Research has shown that individuals from stepfamilies tend to have less secure parental attachment than those from intact families, which increases vulnerability to depression (Branje et al. 2010). This is also consistent with the integrative hypothesis, which posits that each parent plays an irreplaceable role in a child's psychological development (Duchesne and Ratelle 2014). Nevertheless, this proposition requires further empirical validation, and continued exploration of the relationship between parents and parent‐child attachment is warranted.

Notably, in the case of paternal and maternal attachment incongruence, insecure maternal attachment showed a closer relationship with daughters' depressive symptoms compared to paternal attachment, whereas no significant effect was observed for sons. In the context of Chinese culture, compared to fathers, mothers typically engage in more nuanced emotional communication, which fosters stronger emotional bonds with their children (Van Lissa et al. 2019). Additionally, daughters tend to place greater importance on emotional closeness and are more likely to internalize emotional cues from both parents (Chen et al. 2018; Fingerman et al. 2020). In contrast, sons may be less sensitive to discrepancies in paternal and maternal attachment styles, which could explain why these attachment patterns have a weaker effect on their depressive symptoms compared to daughters (Fingerman et al. 2020). According to Feigt et al. (2021), the association between insecure maternal attachment and daughters' depressive symptoms can be interpreted through the lens of intergenerational transmission of gender role attitudes, emotional expression norms, and responses to gender‐based discrimination (Feigt et al. 2021). Specifically, mothers shaped by restrictive gender norms or sexist experiences may model emotional suppression and relational dependency, while daughters may internalize through observational learning during identity development, thereby heightening their vulnerability to depression (Cordero‐Coma and Esping‐Andersen 2018; Feigt et al. 2021). Importantly, maternal emotional distress has also been shown to negatively affect daughters' attachment security and socio‐emotional development (Cheng and Furnham 2020). In contrast, a secure mother‐daughter attachment may indicate that mothers are better able to understand and respond to their daughters' emotional needs from a shared gender perspective, enabling daughters to receive warmer caregiving (Pinquart 2023). Our findings underscore the central role of mothers in shaping daughters' risk and resilience related to depressive symptoms. Therefore, future family‐based intervention research on depression should focus on improving mothers' own mental health and the quality of maternal attachment to support the development of children's emotion regulation (Paley and Hajal 2022), shape gender‐related coping patterns, and enhance the protective influence of secure maternal attachment, particularly for daughters (Feigt et al. 2021).

This study also found that high levels of peer attachment might moderate the impact of incongruence in paternal and maternal attachment on depressive symptoms among college students. The family‐peer connection theory and protective‐protective model suggest parental and peer influences are not independent; one protective factor can enhance the other (Costa et al. 2005; McGinley and Evans 2020). In the pathway where parental attachment is related to depressive symptoms, both secure parental and peer attachments could protect against depression, reinforcing each other as “double insurance.” Additionally, peer attachment can provide “timely buffering” by reducing risks associated with insecure or inconsistent paternal and maternal attachments (Huang et al. 2024). Notably, as individuals enter early adulthood and begin to balance achievement‐oriented individual roles with relational roles (Bishop et al. 2018; Lewis et al. 2025), the influence of parents tends to decline, while peer relationships become increasingly important for emotional development (Pugliese and Okun 2014). The optimized attachment style (e.g., individuals with insecure parental attachment supported by secure peer bonds) during this transitional period may reduce vulnerability to depression (Cook et al. 2016). Our results support the hypothesis that distinct and complementary patterns may exist among these attachment types (Santiago et al. 2017; Yang et al. 2022), providing valuable guidance for intervention practices. For example, previous research has suggested that peer relationships may serve as an effective platform for emotion‐focused interventions, particularly when traditional family‐based programs are less accessible or less applicable during emerging adulthood (Manvelian et al. 2023).

It is important to acknowledge some limitations in the study. First, the findings relied solely on students' self‐reports, which may not fully capture the complexity of parent‐child dynamics. Future studies should consider including parental reports for a more comprehensive perspective. Second, the cross‐sectional design of this study limits the ability to draw causal inferences. To address this, experimental research or longitudinal studies are necessary. Third, this study examined only peer attachment as a moderating variable. Future research should consider additional factors, such as personality traits (e.g., neuroticism) and broader social support systems, which may also interact with parental attachment in shaping mental health outcomes. Fourth, we acknowledge that our sample is limited to Chinese participants, which means that the generalizability of our conclusions to other cultural contexts should be approached with caution. For example, attachment structures and functions differ significantly between Eastern and Western cultures, with the West centering on romantic partners and focusing on individual agency, while East Asia relies more on pluralistic social networks and emphasizes collectivism (Joo et al. 2025). Overreliance on parents may be considered immature in the West, but in East Asia, it may be a natural choice for maintaining family harmony. Therefore, it is essential to explore cross‐cultural emotional connections from a multi‐dimensional perspective.

In summary, this study emphasizes the role of parental attachment (in)congruence in depressive symptoms of college students, as well as the moderating role of gender differences and peer attachment, thereby advancing knowledge of depression to guide prevention and early intervention.

## Conflicts of Interest

The authors declare no conflicts of interest.

## Supporting information


**Data S1:** Supporting Information

## Data Availability

Research data are not shared.
