# Supplementary material for: The Relationship Between Parent‐Child Attachment and Peer Attachment and Depression in College Students: A Moderated Polynomial Regression With Response Surface Analyses
Source: Psych J. 2025 Sep 21;14(5):658–68. doi: 10.1002/pchj.70052 (PMC12520844; doi:10.1002/pchj.70052)
Supplement: Supplementary file 1 — Data S1: Supporting Information [file PCHJ-14-658-s001.docx]

**SUPPLEMENTARY MATERIALS**

**RESULTS**

**Testing for the moderated (in)congruence effects on female students’ depressive symptoms**

As shown in Table S1, the significant ΔR² in the third step of the regression analysis indicates that peer attachment moderated the relationship between parental attachment (in)congruence and depressive symptoms among female students. For clarity, we analyzed the slopes and curvatures of the congruence and incongruence lines at low (-1 *SD* below the mean) and high (1 *SD* above the mean) levels of attachment (see Table S2). A response surface plot illustrating the results of the polynomial regression is presented in Figure S1.

| **TABLE S1** Hierarchical regression predicting female students’ psychological health from paternal-maternal attachment congruency and peer attachment | | | | | | | | |
| --- | --- | --- | --- | --- | --- | --- | --- | --- |
| **Variables** | | **ΔR^2^** | | ***β*** | | ***SE*** | | ***t*** |
| First step: | |  | |  | |  | |  |
| Age | |  | | -0.11^***^ | | 0.03 | | -3.24 |
| Sibling | |  | | -0.02 | | 0.03 | | -0.71 |
| Second step: | | 0.16^***^ | |  | | | | |
| Paternal attachment（X） | |  | | -0.03 | | 0.06 | | -0.50 |
| Maternal attachment（Y） | |  |  | -0.30^***^ | | 0.07 | | -4.52 |
| X^2^ | |  |  | 0.15^**^ | | 0.05 | | 2.87 |
| XY | |  |  | -0.10 | | 0.06 | | -1.60 |
| Y^2^ | |  |  | 0.06 | | 0.04 | | 1.59 |
| Third step: | | 0.03^***^ | |  | | | | |
| Peer attachment（W) | |  | | -0.05 | | 0.05 | | 0.86 |
| WX | |  | | -0.04 | | 0.07 | | -0.56 |
| WY | |  |  | -0.04 | | 0.07 | | -0.58 |
| WX^2^ | |  |  | -0.14^***^ | | 0.04 | | -3.20 |
| WXY | |  |  | 0.10 | | 0.06 | | 1.83 |
| WY^2^ | |  |  | 0.04 | | 0.04 | | 1.07 |
| R^2^ | | 0.21^***^ | | | | | | |
| *F* | | 15.43^***^ | | | | | | |
| *n*=756，^*^*p* < 0.05, ^**^*p* < 0.01, ^***^*p* < 0.001 | | | | | | | | |
| **TABLE S2** Slopes and curvatures of the in/congruence lines at low/high levels of peer attachment among female college students. | | | | | | | | |
| **Depressive symptoms** | **Congruence line (X = Y)**  **Slope a_1_** | | **Congruence line (X = Y)**  **Curvature a_2_** | | **Incongruence line (X = -Y)**  **Slope a_3_** | | **Incongruence line (X = -Y) Curvature a_4_** | |
| Low peer attachment  (-1 *SD*) | -0.25^**^ | | 0.11^*^ | | 0.27 | | 0.50^***^ | |
| High peer attachment  (+1 *SD*) | -0.41^***^ | | 0.12^*^ | | 0.27 | | 0.11 | |
| *n*=756，^*^ *p*＜0.05，^**^ *p*＜0.01，^***^ *p*＜0.001 | | | | | | | | |

1. Female group - Low peer attachment

**
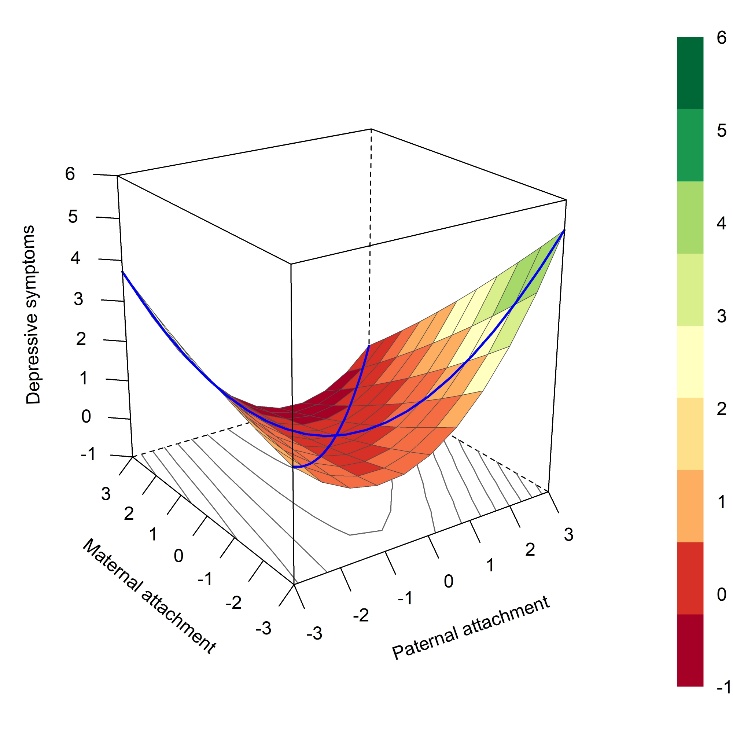
**

1. Female group - High peer attachment

**
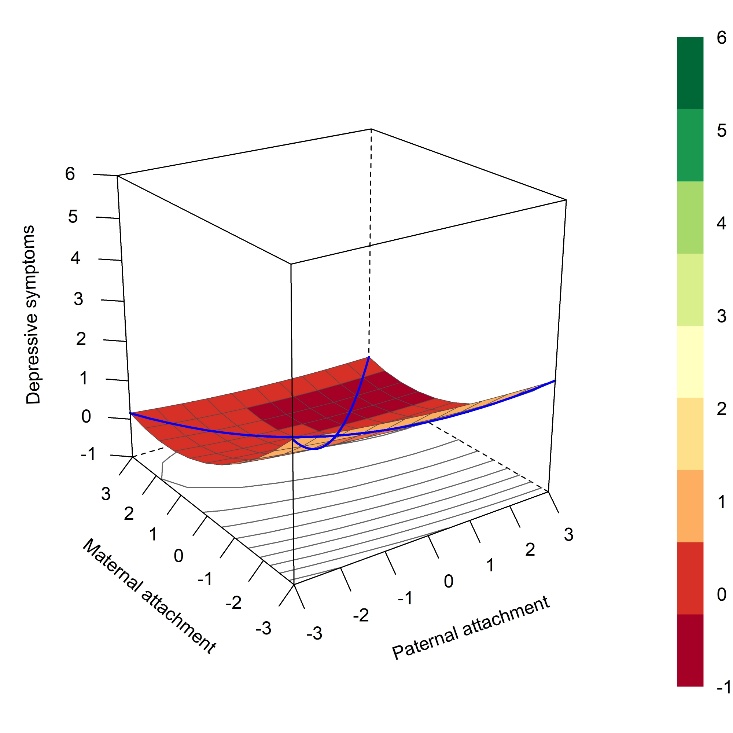
**

**FIGURE S1** Response surface plots for female college students. (A) plot of the low peer attachment group. (B) plot of the high peer attachment group. The rotation position is x = −63, y = 32, z = 15; The color in the response surface indicates the level of outcomes.

**Testing for the moderated (in)congruence effects on male students’ depressive symptoms**

As shown in Table S3, the significant ΔR² in the third step of the regression analysis indicates that peer attachment moderated the relationship between parental attachment (in)congruence and depressive symptoms among male students. For clarity, we analyzed the slopes and curvatures of the congruence and incongruence lines at low (-1 *SD* below the mean) and high (1 *SD* above the mean) levels of attachment (see Table S4). A response surface plot illustrating the results of the polynomial regression is presented in Figure S2.

| **TABLE S3** Hierarchical regression predicting male students’ psychological health from paternal-maternal attachment congruency and peer attachment | | | | | | | | |
| --- | --- | --- | --- | --- | --- | --- | --- | --- |
| **Variables** | | **ΔR^2^** | | ***β*** | | ***SE*** | | ***t*** |
| First step: | |  | |  | |  | |  |
| Age | |  | | 0.02 | | 0.03 | | 0.53 |
| Sibling | |  | | -0.06 | | 0.03 | | -1.72 |
| Second step: | | 0.08^***^ | |  | | | | |
| Paternal attachment（X） | |  | | -0.19^*^ | | 0.07 | | -2.57 |
| Maternal attachment（Y） | |  |  | -0.30^***^ | | 0.07 | | -4.16 |
| X^2^ | |  |  | 0.07 | | 0.06 | | 1.19 |
| XY | |  |  | -0.05 | | 0.09 | | 0.51 |
| Y^2^ | |  |  | 0.19^**^ | | 0.07 | | 2.79 |
| Third step: | | 0.06^***^ | |  | | | | |
| Peer attachment（W) | |  | | 0.02 | | 0.07 | | 0.26 |
| WX | |  | | -0.21^***^ | | 0.07 | | -3.24 |
| WY | |  |  | -0.10 | | 0.07 | | -1.42 |
| WX^2^ | |  |  | -0.04^***^ | | 0.05 | | -0.88 |
| WXY | |  |  | 0.23^***^ | | 0.07 | | 3.34 |
| WY^2^ | |  |  | -0.03 | | 0.05 | | -0.56 |
| R^2^ | | 0.14^***^ | | | | | | |
| *F* | | 10.13^***^ | | | | | | |
| *n*=808，^*^*p* < 0.05, ^**^*p* < 0.01, ^***^*p* < 0.001 | | | | | | | | |
| **TABLE S4** Slopes and curvatures of the in/congruence lines at low/high levels of peer attachment among male college students. | | | | | | | | |
| **Depressive symptoms** | **Congruence line (X = Y)**  **Slope a_1_** | | **Congruence line (X = Y)**  **Curvature a_2_** | | **Incongruence line (X = -Y)**  **Slope a_3_** | | **Incongruence line (X = -Y) Curvature a_4_** | |
| Low peer attachment  (-1 *SD*) | -0.17^**^ | | 0.14^*^ | | 0.23 | | 0.51 | |
| High peer attachment  (+1 *SD*) | -0.80^***^ | | 0.46^***^ | | 0.00 | | -0.10 | |
| *n*=808，^*^ *p*＜0.05，^**^ *p*＜0.01，^***^ *p*＜0.001 | | | | | | | | |

1. Male group - Low peer attachment

**
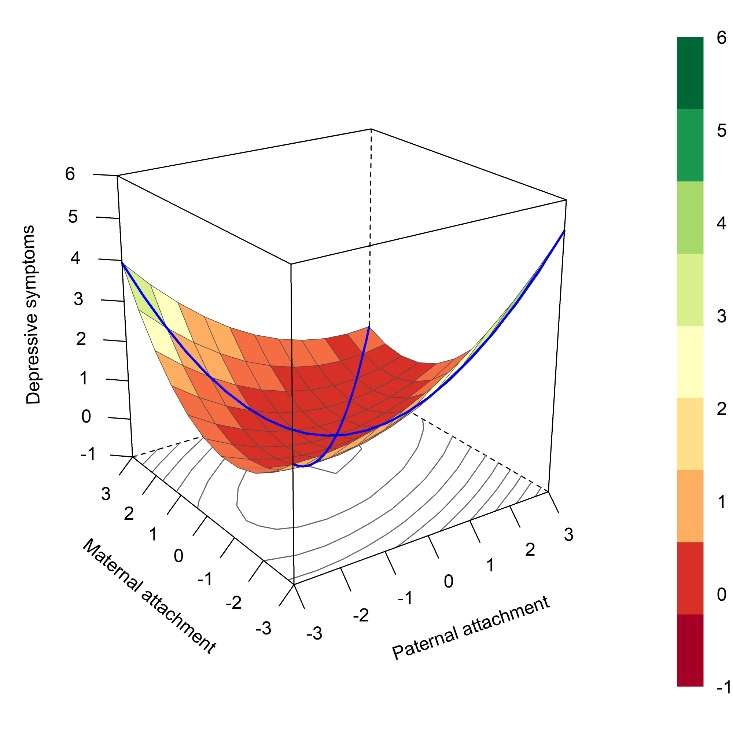
**

1. Male group - High peer attachment

**
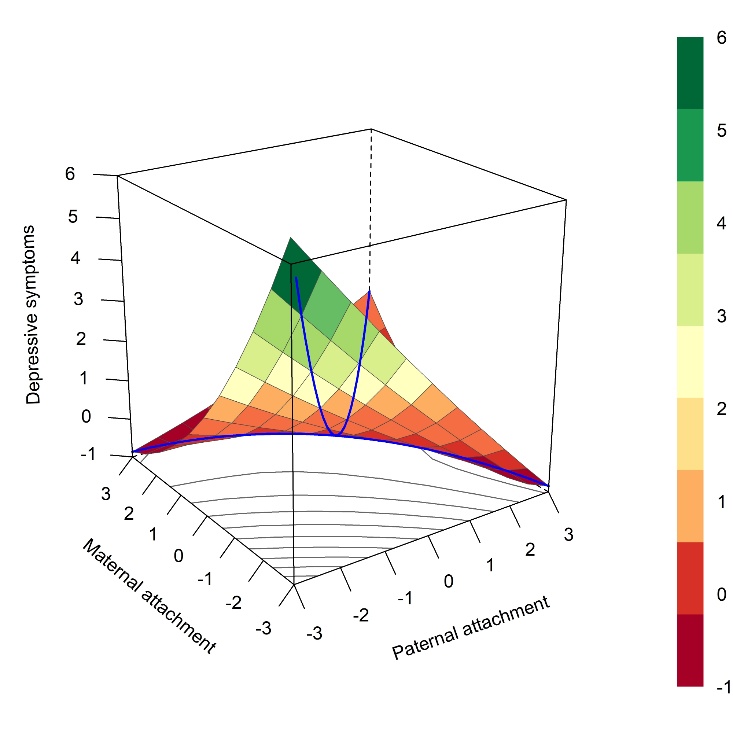
**

**FIGURE S2** Response surface plots for male college students. (A) plot of the low peer attachment group. (B) plot of the high peer attachment group. The rotation position is x = −63, y = 32, z = 15; The color in the response surface indicates the level of outcomes.

Furthermore, we performed a linear regression with parental-peer attachment as the independent variable and depressive mood as the dependent variable, from which we obtained the Mahalanobis distance for each data point. By applying a significance threshold of *p* < .001 and the corresponding χ² value, we identified and removed 13 outliers from our dataset that could potentially distort the analysis. We then retested the moderated (in)congruence effects on male students’ depressive symptoms (see Tables S3a, 4a, and Figure S2a).

| **TABLE S3a** Hierarchical regression predicting male students’ psychological health from paternal-maternal attachment congruency and peer attachment after excluding 13 outliers. | | | | | | | | |
| --- | --- | --- | --- | --- | --- | --- | --- | --- |
| **Variables** | | **ΔR^2^** | | ***β*** | | ***SE*** | | ***t*** |
| First step: | |  | |  | |  | |  |
| Age | |  | | 0.02 | | 0.03 | | 0.53 |
| Sibling | |  | | -0.05 | | 0.03 | | -1.47 |
| Second step: | | 0.08^***^ | |  | | | | |
| Paternal attachment（X） | |  | | -0.18^*^ | | 0.08 | | -2.14 |
| Maternal attachment（Y） | |  |  | -0.31^***^ | | 0.08 | | -3.73 |
| X^2^ | |  |  | 0.05 | | 0.10 | | 0.54 |
| XY | |  |  | 0.03 | | 0.14 | | 0.23 |
| Y^2^ | |  |  | 0.25^**^ | | 0.10 | | 2.60 |
| Third step: | | 0.06^***^ | |  | | | | |
| Peer attachment（W) | |  | | 0.01 | | 0.07 | | 0.08 |
| WX | |  | | -0.15 | | 0.10 | | -1.52 |
| WY | |  |  | -0.18 | | 0.10 | | -1.82 |
| WX^2^ | |  |  | -0.01 | | 0.10 | | -0.11 |
| WXY | |  |  | 0.15 | | 0.17 | | 0.90 |
| WY^2^ | |  |  | 0.02 | | 0.11 | | 0.17 |
| R^2^ | | 0.14^***^ | | | | | | |
| *F* | | 9.62^***^ | | | | | | |
| *n*=795，^*^*p* < 0.05, ^**^*p* < 0.01, ^***^*p* < 0.001 | | | | | | | | |
| **TABLE S4a** Slopes and curvatures of the in/congruence lines at low/high levels of peer attachment among male college students after excluding 13 outliers. | | | | | | | | |
| **Depressive symptoms** | **Congruence line (X = Y)**  **Slope a_1_** | | **Congruence line (X = Y)**  **Curvature a_2_** | | **Incongruence line (X = -Y)**  **Slope a_3_** | | **Incongruence line (X = -Y) Curvature a_4_** | |
| Low peer attachment  (-1 *SD*) | -0.15^*^ | | 0.18^**^ | | 0.10 | | 0.41 | |
| High peer attachment  (+1 *SD*) | -0.82^***^ | | 0.49^***^ | | 0.15 | | 0.13 | |
| *n*=795，^*^ *p*＜0.05，^**^ *p*＜0.01，^***^ *p*＜0.001 | | | | | | | | |

（A）Male group - Low peer attachment


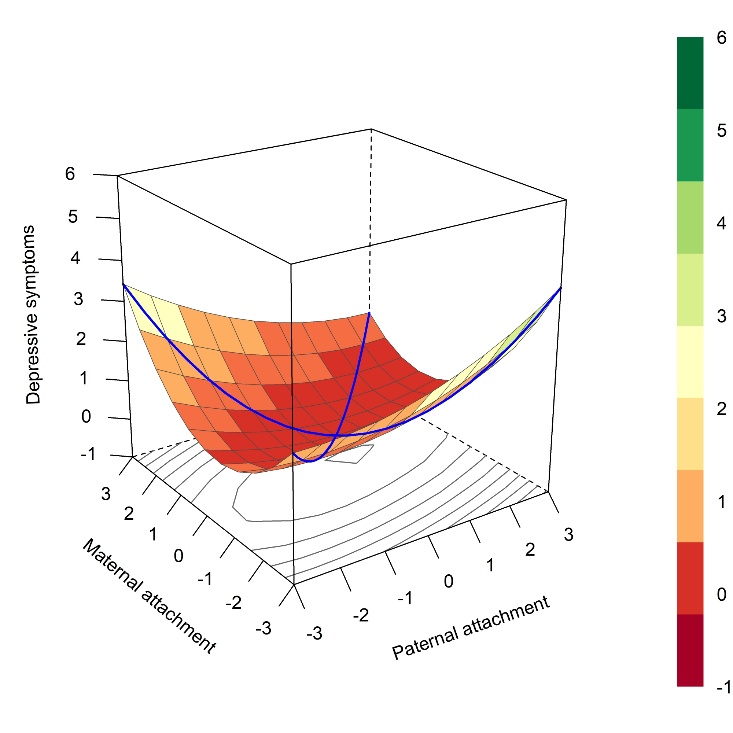


（B）Male group - High peer attachment


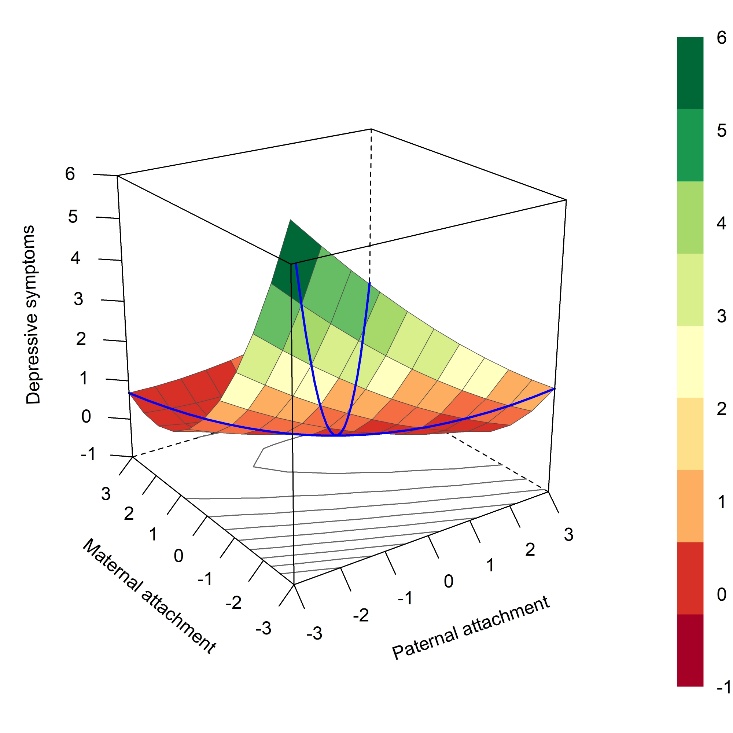


**FIGURE S2a** Response surface plots for male college students after excluding 13 outliers. (A) Plot of the low peer attachment group. (B) Plot of the high peer attachment group. The rotation position is x = −63, y = 32, z = 15; The color in the response surface indicates the level of outcomes.
